# A novel scoring system for predicting disease severity without CT imaging in acute diverticulitis

**DOI:** 10.1007/s00384-024-04740-6

**Published:** 2024-10-15

**Authors:** Leena-Mari Mäntymäki, Juha Grönroos, Jukka Karvonen, Mika Ukkonen

**Affiliations:** 1 Department of Surgery, University of Turku, Turku, Finland; 2 Department of Gastroenterology and Alimentary Tract Surgery, Tampere University Hospital, Tampere, Finland; 3 Department of Digestive Surgery, Turku University Hospital, Turku, Finland; 4 Department of Surgery, University of Tampere, Tampere, Finland

**Keywords:** Uncomplicated acute diverticulitis, Complicated acute diverticulitis, Computed tomography, Clinical scoring

## Abstract

**Purpose:**

Clinical scoring could help physicians identify patients with suspected acute diverticulitis who would benefit from further evaluation using computed tomography imaging. The aim of the study was to identify risk factors for complicated acute diverticulitis and create a risk score to predict disease severity in acute diverticulitis.

**Methods:**

Patients diagnosed with CT-verified acute diverticulitis between 2015 and 2017 were included. Data on patients’ clinical and laboratory findings and medical histories were collected retrospectively. Risk factors for complicated acute diverticulitis were identified using univariate and multivariate analyses. Continuous laboratory values were categorised by cut-off points determined using receiver operating characteristic (ROC) analysis. The Acute Diverticulitis Severity Score was formulated using logistic regression analysis.

**Results:**

Of the total 513 patients included in the study, 449 (88%) had UAD, and 64 (12%) had CAD. Older age, significant comorbidities, C-reactive protein level, leucocyte count, vomiting, and body temperature were found to be independently associated with a higher risk for CAD. The novel Acute Diverticulitis Severity Score could reliably detect patients with CAD. The area under the ROC curve was 0.856 (*p* < 0.001) in discriminating disease severity. While higher scores indicate radiological studies, patients with low scores face an almost non-existent risk for complicated disease, making such studies possibly redundant.

**Conclusions:**

The Acute Diverticulitis Severity Score accurately separated patients with uncomplicated disease from those at risk for complicated disease. This score can be applied in daily clinical practice to select patients requiring further investigation, consequently reducing healthcare costs and burdens.

## Background

In recent decades, the incidence of acute diverticulitis (AD) has increased, contributing to escalating healthcare costs associated with diagnosis and treatment [1, 2]. Most patients present with uncomplicated acute diverticulitis (UAD), which is considered a self-limiting condition that does not necessarily require antibiotic treatment or operative management [3, 4]. Conversely, complicated acute diverticulitis (CAD) requires often hospitalisation, antibiotics, further colonic investigations, or, in some cases, even surgery. Thus, it is important to differentiate between these two forms of AD to select the optimal treatment for each patient.

The diagnosis and severity assessment of AD is commonly based on CT imaging, known for its high specificity and sensitivity [5]. Given the challenges of clinically diagnosing AD, even for experienced physicians [6, 7], many guidelines suggest CT imaging for diagnosis [8, 9]. Nevertheless, CAD is associated with several risk factors, including high levels of C-reactive protein (CRP) and leucocytes, certain clinical signs, an initial AD episode, and steroid use [10]. However, relying solely on individual risk factors is considered insufficient to predict the severity of AD. Consequently, different prognostic models or clinical scoring systems for AD have been proposed [11, 12]. Clinical scoring systems are already employed in acute appendicitis, and some guidelines suggest the use of clinical scoring systems to identify patients who would benefit the most from CT imaging [13, 14]. However, a similar practical, easy-to-use, and reliable diagnostic scoring system for AD has not yet been widely established in daily clinical practice. More accurate clinical detection of probable UAD could reduce patients’ referrals to secondary care and reliance on CT imaging, potentially lowering healthcare costs and patient exposure to ionising radiation.

Thus, our aim was to identify risk factors for CAD and create an easy-to-use clinical scoring system that could predict disease severity, thus assisting physicians in determining the need for further patient evaluation.

## Methods

A retrospective cohort study was conducted in a tertiary referral hospital, where approximately 250 cases of AD are diagnosed annually in the emergency department. All consecutive patients with CT-verified colonic AD in 2015–2017 were included in the study. All patients underwent intravenous contrast-enhanced CT scans (Siemens Somatom Definition, Siemens Healthcare Diagnostics AB). These scans were analysed by in-house radiologists available 24/7, including residents or consultants, and were later reanalysed by consultant body radiologists specialised in body CT scans. Patients whose diagnoses changed after the emergency department visit, even if the CT report remained consistent, were excluded from the analysis. Also, patients with missing data were excluded.

We manually collected relevant data from electronic patient charts, including age, sex, comorbidities, medications, clinical and initial laboratory findings at index admission, duration of symptoms, and CT findings. Disease severity was classified as uncomplicated or complicated based on CT scans and clinical status according to the classification published by Sallinen et al. [15]. The patients were diagnosed with UAD in the presence of colon wall thickening and pericolic inflammatory changes, consistent with stage 1 diverticulitis. CAD was identified by the occurrence of an abscess or perforation with or without peritonitis or organ dysfunction, qualifying as stage 2 diverticulitis or higher.

### Acute diverticulitis severity score

The diagnostic score was constructed by logistic regression analysis. First, variables associated with a higher risk for CAD identified through univariate analysis were selected for multivariate analysis. Then, variables independently related to CAD were incorporated into the score. Although cardiac comorbidities were linked to CAD in the univariate analysis, their lack of association in the multivariate analysis led to its exclusion. Instead, we used a new variable that incorporated any significant comorbidity. Continuous laboratory values, including leukocyte count, CRP level, and body temperature, were categorised according to cut-off points determined using receiver operating characteristic (ROC) analysis. The cut-off points were (1) the value corresponding to 80% sensitivity, (2) that corresponding to 80% specificity, and (3) the mean of these two values. The cut-off values for the CRP level were rounded to the nearest 5 units. Because the distribution of the CRP values differed in patients with symptoms lasting less than or more than 24 h, two cut-off points were used for CRP. The score points were obtained from regression coefficients rounded to the nearest integer. Since patients with peritonitis or sepsis require radiological investigations, they were given the maximum number of points, reflecting the necessity of CT imaging in these patients. Correspondingly, those with ongoing wide-spectrum antibiotic therapy were also considered to require radiological examinations and were given the maximum number of points. Patients with missing data were excluded from the analysis.

### Statistical analysis

Statistical analysis was performed using SPSS version 22. The *χ*^2^ test or Fisher’s exact test was performed to compare categorical variables, and the Mann–Whitney *U*-test was employed for continuous variables. Continuous variables are presented as means (and standard deviation) or medians (along with the minimum and maximum values or the interquartile range), depending on the value distribution. Regression analysis was used to identify independently associated risk factors. Subsequently, we determined the cut-off points of the continuous variables through ROC analysis. The Kruskal–Wallis test was applied to estimate the statistical significance of the categorised variables. Finally, the regression coefficients were calculated using logistic regression analysis employing the enter method. A *p*-value < 0.05 was considered statistically significant.

### Ethical aspects

The study protocol was approved by the local ethics committee. Informed consent from the patients was not required.

## Results

A total of 513 patients (median age 59 [28–96] years; 64% female) were included in the study, of whom 449 (88%) had UAD and 64 (12%) had CAD. The univariate analysis of patient-related characteristics revealed that only chronic cardiovascular disease (*p* = 0.011), any significant pre-existing comorbidity (*p* = 0.007), and older age (*p* < 0.001) were predictors of CAD risk. Clinical peritonitis occurred in 6 (1.1%) patients and abscess in 47 (9.2%) patients. The patient demographics are shown in Table 1.

**Table 1:** Patient characteristics

Variable	UAD n = 449	CAD *n* = 64	*p*-value
Age, years, range	58 (28–94)	67 (34–96)	< 0.001
Age ≥ 60 years	202 (45%)	46 (69%)	< 0.001
Sex, female/male	284/165 (63/37%)	43/21 (67/33%)	0.540
Comorbidities			
Cardiac disease	164 (37%)	34 (53%)	0.011
Pulmonary disease	41 (9%)	8 (13%)	0.391
Diabetes	36 (8%)	9 (14%)	0.110
Rheumatoid disease	15 (3%)	3 (5%)	0.394
Neurological disease	12 (3%)	3 (5%)	0.285
Kidney disease	7 (2%)	0 (0%)	0.391
Inflammatory bowel disease	7 (2%)	0 (0%)	0.391
Liver disease	2 (1%)	1 (2%)	0.330
Malignancy	8 (2%)	1 (2%)	0.688
Any significant comorbidity	221 (49%)	43 (67%)	0.007
Corticosteroid medication	21 (5%)	4 (6%)	0.382
Earlier diverticulitis	112 (25%)	20 (31%)	0.280
CRP, mg/L, IQR	101 (61–150)	166 (99–221)	< 0.001
Leukocyte count, × 109 mg/L,IQR	10.8 (8.9–13.0)	12.5 (9.6–15.2)	0.002
Mean arterial pressure, IQR	104 (96–114)	103 (92–111)	0.239
Body temperature, °C, IQR	37.1 (36.6–37.5)	37.2 (36.8–37.6)	0.046
$\ge$37.0 °C	253 (56%)	46 (72%)	0.018
$\ge$38.5 °C	20 (5%)	3 (5%)	0.565
Vomiting	14 (3%)	9 (14%)	< 0.001
Peritonitis	0 (0.0%)	6 (9%)	< 0.001
Localization of the pain			
Left lower abdomen	63 (14%)	13 (20%)	0.285
Lower abdomen	229 (51%)	31 (49%)	
Other/diffuse	157 (35%)	20 (31%)	

*CRP* c-reactive protein, *IQR* interquartile range, data presented as median with (interquartile range), *UAD* uncomplicated acute diverticulitis,

*CAD* complicated acute diverticulitis

Through multivariate analysis, we found six factors independently associated with increased CAD risk: older age (*p* < 0.001, OR 1.054, 95% CI 1.027–1.081), significant pre-existing comorbidity (*p* = 0.043, OR 1.066, 95% CI 0.543–2.092), elevated leucocyte count (*p* = 0.018, OR 1.104, 95% CI 1.017–1.198), increased CRP levels (*p* < 0.001, OR 1.008, 95% CI 1.004–1.011), body temperature (*p* = 0.046, OR 1.686, 95% CI 0.880–3.224), and vomiting (*p* = 0.026, OR 3.151, 95% CI 1.151–8.627). The optimal cut-off values for this predictive score were determined through ROC curve analysis and are provided in Table 2. The corresponding CAD risk estimates for these subgroups are also presented in Table 2.

**Table 2:** Acute diverticulitis severity score

Score	Regression coefficient	Score
Age ≥ 60 years	3.043	3
Significant comorbidity	1.452	1
CRP, mg/L (symptoms less than 1 day)		
>150 (150)	5.031	5
125–149 (80–149)	3.279	3
100–124 (10–79)	1.265	1
< 100 (< 10)	-	0
Body temperature, ≥ 37.0 °C	1.516	2
Leucocytosis, × 10^9^ mg/L		
$\ge$ 13.3	1.942	2
11.3–13.29	0.996	1
9.3–11.29	0.896	1
$\le$ 9.3	-	0
Vomiting	3.106	3
Peritonitis or sepsis	-	Max value
Worsening symptoms despite ongoing wide-spectrum antibiotics	-	Max value

*CRP* c-reactive protein

**Fig. 1:**
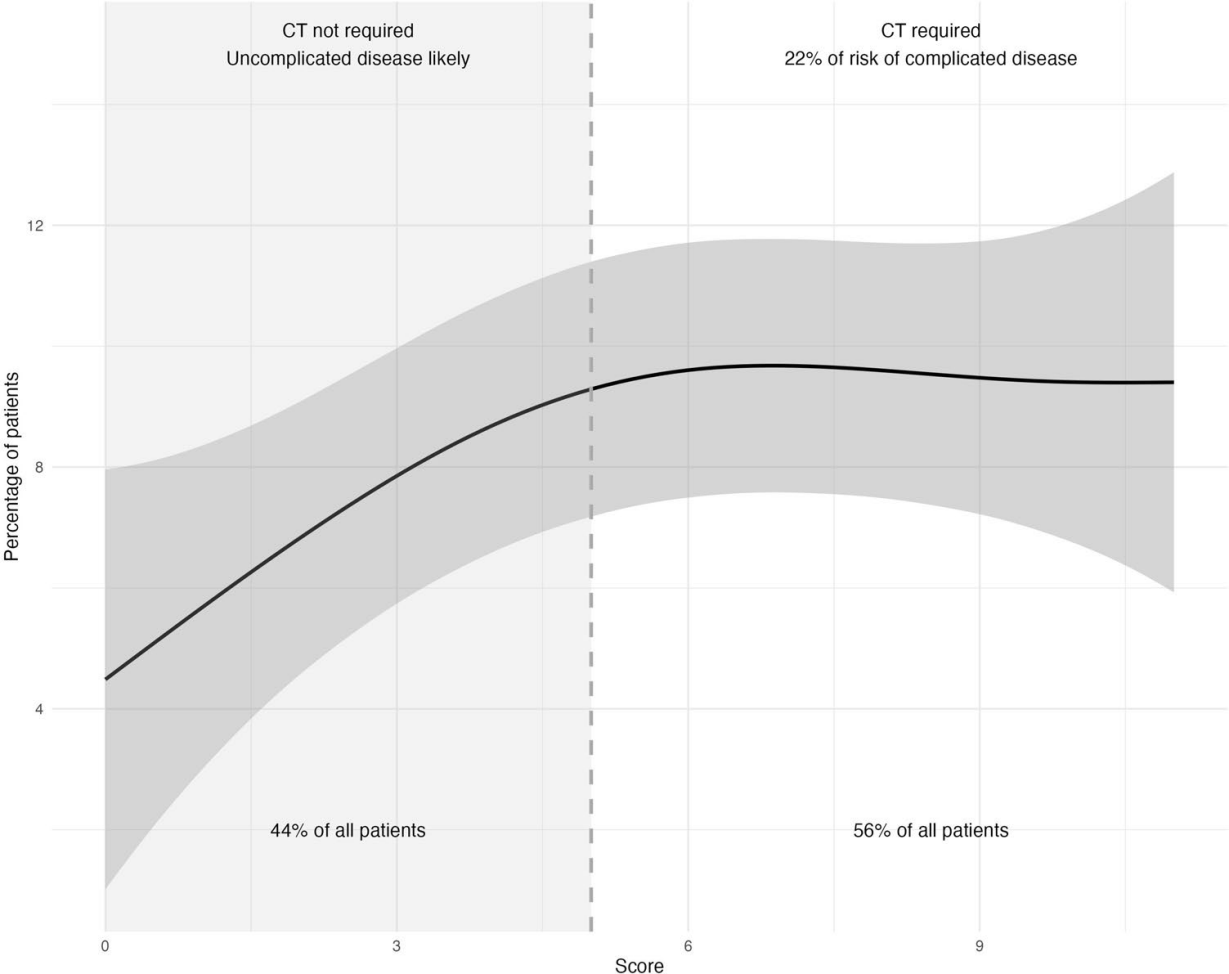
Share of patients with different score points and risk of CAD

Further examination revealed that patients with scores of five or less had a very low risk for CAD, as shown in Table 3 and Fig. 1. In a single case with a low score, the radiologist considered the possibility of either a small abscess or an inflamed diverticulum. Given the ambiguous CT findings, we classified this patient into the CAD group. Following the clinician’s assessment, this patient’s treatment followed UAD protocols, leading to an uneventful recovery. However, a significant increase in risk was observed when the score exceeded 10 points.

**Table 3:** Share of patients with uncomplicated and complicated acute diverticulitis by scores

Score	UAD	CAD
	*n* = 449	*n* = 64
0	12 (100%)	0 (0.0%)
1	29 (100%)	0 (0.0%)
2	31 (100%)	0 (0.0%)
3	36 (100%)	0 (0.0%)
4	61 (100%)	0 (0.0%)
5	52 (98%)	1* (1.9%)
6	57 (90%)	6 (9.5%)
7	50 (91%)	5 (9.1%)
8	35 (83%)	7 (17%)
9	32 (76%)	10 (24%)
10	18 (82%)	4 (18%)
> 10	36 (53%)	31 (47%)
0–5	221 (99.5%)	1* (0.5%)
6–10	192 (86%)	32 (14%)
> 10	36 (54%)	31 (46%)

^*^Either a small abscess or inflamed diverticula, treated as a UAD

*UAD* uncomplicated acute diverticulitis, *CAD* complicated acute diverticulitis

**Fig. 2:**
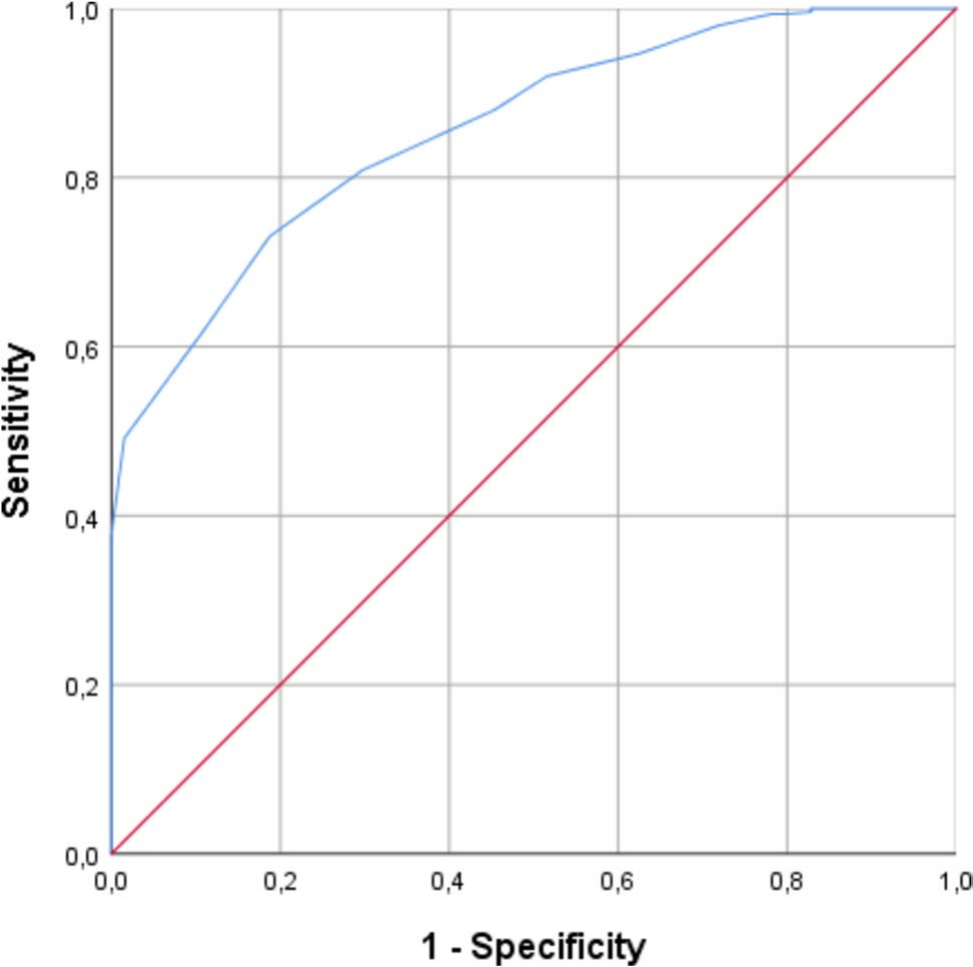
ROC curve of the new score in detecting uncomplicated acute diverticulitis

The score was subjected to ROC analysis, yielding an excellent discrimination level (AUC 0.856, *p* < 0.001), as illustrated in Fig. 2. While patients with scores above five had a risk for CAD ranging from 9.1 to 47%, the score effectively identified low-risk patients, eliminating the need for further examinations.

## Discussion

Although CT imaging is recommended for diagnosing AD, it exposes patients to radiation, requires resources, and generates healthcare costs. To address these concerns, we created a disease severity score for predicting the severity of AD designed to assist clinicians in identifying patients who would benefit from referral to secondary care and further CT imaging. With our scoring system, patients with UAD can be distinguished from CAD patients requiring further investigations and management.

In this study, older age and high CRP and leucocyte levels, body temperature, presence of vomiting, and comorbidities were associated with a higher risk of CAD. Generally, older patients benefit more frequently from CT imaging than younger patients [16], with CT being advised to confirm AD diagnoses and distinguish CAD from UAD in older adults [17]. Elevated CRP levels have been found to be a risk factor for CAD [10]. Nonetheless, the optimal thresholds have been shown to vary among studies. According to Mäkelä et al. [18, 19], CRP values of 149.5 mg/L or more effectively distinguished between uncomplicated and complicated diverticulitis; however, low CRP values did not reliably predict UAD. Another study identified 175 mg/L as the optimal CRP threshold for discriminating between UAD and CAD, noting a 15% risk of CAD for CRP levels less than 25 mg/L [20]. Since the optimal threshold of CRP levels varies, we stratified the risk points according to the CRP levels. Notably, the CRP levels may be misleadingly low among those with symptoms of less than 12 h. Therefore, we adjusted the CRP levels incorporated into the risk scores, differentiating patients with symptoms occurring less than and over 1 day before presentation. High leucocyte count has also been proposed as a risk factor for CAD [21], although the optimal threshold is unclear. Our scoring system attempts to stratify the risk according to leucocyte count levels. Fever, a classic symptom of AD, is also considered an independent risk factor for CAD [10]. In a study by van de Wall et al. [20], body temperature showed no significant difference between patients with UAD and CAD. In our study, higher body temperature was significantly associated with CAD. However, our data did not account for potential confounding factors, such as antipyretic use. In addition to fever, vomiting emerged as a significant risk factor for CAD, which aligns with earlier findings [20]. Retrospective cohorts not stratified by disease severity have suggested that the absence of vomiting could be a predictive marker of AD [7, 22]. Left lower quadrant pain, another hallmark symptom of AD [23], did not significantly discriminate between UAD and CAD in our study nor did any other location of the pain. Compared to other studies that included patients with suspected AD, our study reported a lower incidence of patients with only left lower quadrant pain [22, 24]. At the same time, it has been found that CAD patients have more diffuse abdominal pain than patients with UAD [20]. All patients with clinical peritonitis in our study had CAD.

Various scoring systems have been introduced to enhance clinical accuracy in diagnosing AD. Andeweg et al. [22] proposed a nomogram based on several individual predictors of AD. In a prospective study, Lameris et al. [7] developed a decision rule based on three variables: tenderness only in the left lower quadrant of the abdomen, absence of vomiting, and a CRP level above 50 mg/L. Although both studies proposed clinical scoring systems to improve the clinical diagnosis of AD, they did not differentiate between UAD and CAD. Another recent study by Sigurdardottir et al. [24] formulated a risk score to predict the probability of acute diverticulitis, which can be used on a website. A retrospective study conducted by Bolkenstein et al. [11] on 943 AD patients, with 18% experiencing CAD, found several individual risk factors for CAD. After validating their diagnostic model, the authors showed that CRP, leucocyte count, and abdominal guarding were significantly related to CAD. Next, the CRP and leucocyte levels were divided into different categories to indicate varying CAD risk levels. Covino et al. [12] created the PACO-D score to increase clinical accuracy in diagnosing AD. In their study, patients were categorised into three different risk classes. The variables selected for their score from the multivariate analysis included sex (male), constipation, not using proton pump inhibitor medication, Hb levels below 11.9 g/L, CRP levels above 80 mg/L, and obesity (BMI > 30). Each variable accounted for 1 point, and the high risk for CAD was ≥ 4 points. One major difference in our study compared to previous work on scoring systems lies in how we classified the severity of AD. For example, Bolkenstein et al. [11] used the Hinchey classification, while Sigurdardottir et al. [24] defined AD as complicated if CT imaging showed perforation, including pericolic extraluminal gas. Covino et al. [12] also included patients with diverticular bleeding. Some classifications of AD define pericolic air bubbles as CAD [25, 26]. We selected the classification introduced by Sallinen et al. [15] since it takes into account patients’ clinical, physiological, and radiological findings. In this classification, pericolic air bubbles seen in CT scans indicate UAD. It has also been shown that pericolic air bubbles or small abscesses do not necessarily lead to poor outcomes [15, 27]. In our study, only one patient with CAD had a score < 5. In a single case with a low score, the radiologist considered the possibility of either a small abscess or an inflamed diverticulum. The patient had antibiotic treatment and recovered without intervention or readmission. In addition, it has been shown that outpatient management or treatment without antibiotics is feasible and safe in some CAD with small abscesses or small amounts of extra-luminal air [28, 29].

Some aspects should be considered if the diagnosis of AD is conducted without CT imaging. Numerous conditions can mimic AD, with studies showing that clinical evaluation alone has poor diagnostic accuracy in cases of non-traumatic abdominal pain [6]. Incorrect diagnosis can lead to non-optimal treatment, possible poor outcomes, and increased healthcare costs [16]. However, routine CT imaging for every patient increases costs and might not be cost-effective [30]. The increasing incidence of AD causes a remarkable economic burden, and it has been suggested that the increased healthcare costs related to AD can be due to the increased use of expensive investigations, such as CT imaging [31]. Even though scoring systems can be valuable tools in predicting the severity of suspected AD, it should be emphasised that patients presenting with ongoing or worsening symptoms should be re-evaluated. Although, it has been shown that non-antibiotic treatment in UAD patients is safe [3, 32, 33]. In addition, outpatient management of patients with UAD and some cases of CAD has shown to be safe and feasible [28, 34]. Outpatient management have shown to reduce treatment costs by 67% [34]. Therefore, an observational strategy for patients with suspected acute diverticulitis without severe symptoms may be feasible and safe.

In addition, patients with recurrent AD often recognise the symptoms of AD themselves. Recurrent AD was not found to be a risk factor for CAD in our study. It has also been shown that most cases of recurrent AD are uncomplicated [35] and can thus be treated safely without antibiotics and in outpatient settings [36, 37]. Patients with recurrent AD are exposed to repeated radiation if CT is performed in every recurrent episode of AD. This is a concern especially when the incidence of acute diverticulitis among younger age groups is increasing [31]**.** Therefore, this scoring system may especially benefit patients with recurrent AD by assisting primary care physicians in deciding whether a patient needs to be referred to the emergency department for further diagnostic studies.

This study has some limitations in addition to those mentioned above in the discussion. Due to its retrospective design, some previously suggested risk factors or parameters could not be reliably recorded. Nevertheless, our study’s cohort was large, and the dataset was relatively comprehensive, despite the lack of data on a few patients. Notably, our new disease severity score for AD, although not yet validated, holds potential for future prospective testing. Validation of the score in a prospective multicentre study will improve the reliability of the score.

Our novel Acute Diverticulitis Severity Score could distinguish between patients with uncomplicated disease and those at risk for complicated disease. The score could be a valuable tool for primary healthcare physicians in identifying patients with clinically suspected acute diverticulitis who need to be referred for secondary care. By utilising the score, the need for referrals and, consequently, the need for CT imaging could be decreased leading to reduced healthcare costs.

## Data Availability

No datasets were generated or analysed during the current study.
